# Children’s Understanding of No Diving Warning Signs: Implications for Preventing Childhood Injury

**DOI:** 10.3390/ijerph13070669

**Published:** 2016-07-07

**Authors:** Barbara A. Morrongiello, Amanda Cox, Rachel Scott, Sarah E. Sutey

**Affiliations:** Psychology Department, University of Guelph, Guelph, ON N1G 2W1, Canada; acox01@uoguelph.ca (A.C.); rscott0370@gmail.com (R.S.); ssutey@uoguelph.ca (S.E.S.)

**Keywords:** children, unintentional injury, diving, warning signs

## Abstract

The current study examined children’s understanding of No Diving warning signs. Normally-developing 7 to 10 year olds were asked questions to assess their understanding of text, images, and main messages on No Diving warning signs. These structured interviews were audio recorded and responses were later coded. Results revealed that children understood the behavior advised against (diving), why it is prohibited (can hit head on the bottom), and what can happen (serious injury including hospitalization). They understood that breaking your neck results in limitations in mobility and can occur from diving, but they did not anticipate that such an injury is likely to occur. There were no gender and few age differences, but diving experience was associated with children significantly downplaying their risk of injury. The findings suggest that having No Diving warning signs explicitly mention a broken neck, may serve to remind children of this potential consequence at the time of decision making. Active adult supervision is particularly important for children who have prior positive diving experiences.

## 1. Introduction

Unintentional injuries are the leading cause of death for children in the United States, Canada, and most developed nations [1,2]. A child’s developmental level affects both the type of injury they are vulnerable to and the location where injuries are likely to occur. For young children under 5 years, falls and poisoning injuries are common and typically occur in the home [1]. At these young ages, caregivers’ behaviors are key determinants of injury risk and, therefore, are essential targets for intervention in injury prevention initiatives [3,4]. For school-aged children, pedestrian and cycling injuries are common and most injuries occur when youth are away from home and not being directly supervised when making decisions about engagement in injury-risk activities [5,6,7]. Because their own behavioral choices increasingly come into play to affect school-age children’s risk of injury, prevention efforts need to consider children’s knowledge of hazards and understanding of injury risks. One common approach to alerting people to hazards and injury-risk behaviors is through the posting of warning signs. Of course, whether warning signs are effective for these purposes depends in part on how well viewers understand the intended message [8]. The current study addressed this issue with respect to children and their understanding of various features of No Diving warning signs.

Diving related injuries are often catastrophic and result in life altering effects for victims and their families [9]. Most diving injuries are cervical and result in tetraplegia or quadriplegia rather than paraplegia [10]. Recent research has identified the lifetime financial cost of someone living with quadriplegia to range from 1 to 3 million dollars [10]. Even for developing children, diving can cause permanent spinal cord injury, with estimates ranging from 8% to 33% [11]. Diving incidents during childhood most often involve those 11–14 years of age, followed by children 5–10 years of age [11]. Given these statistics, the prevention of diving into shallow water is a priority. It is important, therefore, to examine children’s knowledge of the risks associated with diving into shallow water and the extent to which they understand typical warning signs that are posted to advise against such behavior.

### 1.1. Warning Signs

Warning signs are typically posted in areas where individuals are likely to get injured, with the goal being to alert, inform, and/or remind individuals of potential hazards and unsafe behaviours that should be avoided. Not surprisingly, for warning signs to be effective the sign must both elicit attention and be understood by viewers [12,13]. Factors that influence a sign’s ability to evoke attention include location, size, font size, color, and border width of the sign [14,15]. Having a large sign located at eye level, in clear view, and proximal to the hazard is best. Using text that is large and presents red letters against a white background has been shown to increase effectiveness in evoking attention; research suggests that the color red may be particularly salient because it is associated with the word “stop” in stop signs and “danger” on poisonous product labels, both of which occur in many cultures worldwide [8].

Research with adults reveals a number of factors that affect viewers’ understanding of the message communicated by a warning sign, include the language expressed (e.g., word choice, explicitness) and pictorial images shown. Including a signal word (e.g., *Caution*, *Warning*, *Danger*) that is immediately understood as communicating something about risk is an essential feature on warning signs [16]. The explicitness of the messaging also affects understanding. Explicit messages are those that give specifics about the hazards (e.g., shallow water), definitive instructions on what not to do (e.g., no diving), and indicate consequences that can occur for not complying [8]. For example, when adults were asked to judge no diving signs that had varying degrees of severity of injury described and depicted in the images, respondents concluded that including the explicit phrase “You could break your neck” was best to communicate the severity of consequences [17].

Including images and pictorial content on warning signs can not only enhance attention but also can communicate vast amounts of information at a glance [8]. Pictorials are typically gender neutral and often appear in black against a white background [18]. When the behavior depicted is encircled and has a slash through it, this is understood as indicating that the character is doing something that is not permitted [13]. Including images has been shown to increase the vividness of warning signs and result in improved memory for the warning, so including a pictorial image on a warning sign is recommended [12,19]. Images also can communicate across language barriers and to the illiterate and are processed more quickly than text, all of which makes the inclusion of images an important addition to warning signs [20].

It is apparent from the extensive research on warning signs that virtually every design detail matters and can affect an individual’s attention to, understanding of, memory for, and compliance with the warning. The majority of this important research, however, has been conducted with adults, even though many warning signs are intended to apply across a broad population and there is a recognized need to identify signs that are effective for children [9]. Addressing this gap, the current study examined children’s understanding of both text and image features on No Diving warning signs. Past research with children is limited not only by virtue of their being few studies but also because the main focus has been on evaluating the effectiveness of posted warning signs for preventing youth diving into pools, rather than assessing youths’ understanding of the sign features per se. These studies found that children in middle school and high school who had prior experience with safely diving into pools (e.g., swim lessons, swim team) engaged in the prohibited behavior regardless of seeing the warning sign, and these effects did not vary with child gender or age [21,22]. Research with adults also has found that prior experience creates expectations that can dampen the impact of warnings on risk behaviors [23]. Although the findings with children indicate that seeing the sign did not ensure compliance once children had diving experience, it is not clear whether children understood the various components that comprised the warning (e.g., signal word, image showing broken neck).

### 1.2. Current Study

The aim of the study was to examine children’s understanding of the typical components of No Diving warning signs, including: signal word (*Danger*, *Caution*, *Warning*), what behavior is defined by “diving”, what a sign saying “No Diving—shallow water” means, what the terms “permanent injury” and “you can break your neck” mean, and what the image (i.e., clipart diver experiencing a broken neck) is intended to communicate. To examine their injury-risk appraisals related to diving, they gave ratings of dangerousness, likelihood of injury, severity of potential injury, and stated whether or not one can wind up in a wheelchair for life from diving. Children 7 to 10 years were tested because this represents an age range in which children increasingly receive less direct supervision and make more autonomous decisions about their activities [24] and at these ages they are at risk for experiencing diving related injuries [10]. In addition to age, gender differences also were considered in examining the results because boys generally experience more diving related injuries than girls [10]; such differential injury rates could indicate boys and girls differ in their understanding of warning signs.

## 2. Method

### 2.1. Participants

Children aged 7 to 10 years were recruited throughout the community in Guelph and the surrounding area (e.g., via posters, newspaper ads) and allocated into either a younger (*N* = 62, 7.0 to 8.5 years, *M* age = 7.52 years, *SD* = 0.68 years) or older (*N* = 58, 8.8 to 10 years, *M* = 9.16 years; *SD* = 0.64 years) group, with equal numbers of boys and girls in each group. All participants were typically developing, as reported by their parents. Nearly all children (95%) had taken formal swim lessons. Generally, parents were unable to report on how long or consistently the child had been in lessons, but they were confident in reporting on whether or not the child had been taught how to dive into the pool and had experience doing so as part of their swim lessons; children confirmed this information during the interview. Of the 120 children, 90 had diving experience (39% or 63% of younger and 51% or 82% of older children), meaning that they had experience diving into open water (e.g., pools, lakes, ocean); no one reported any injuries from doing so. The sample comprised predominantly middle-upper income families (71% earned above $60,000), with 71% of parents having some/completed college/university, and nearly all of the participants Caucasian (98%).

### 2.2. Materials

#### 2.2.1. Photo Sort Task

**Figure 1:**
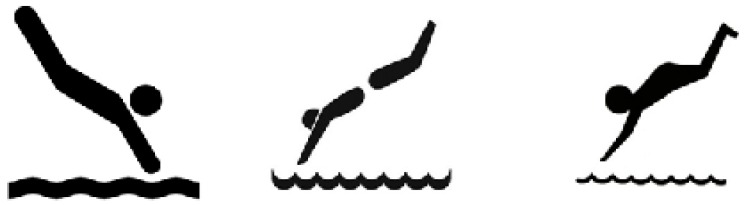
The three clipart images of a figure diving into water that were included in the photo sort task.

To determine children’s understanding of what constitutes a “dive” they were given a deck of 23 image cards (randomized) and asked to sort these into Diving and Not Diving boxes, as they thought best. The deck comprised 20 colored photos of boys and girls who were in a similar age group to the participants (7–10 years old), with the photos showing individual children either diving in a variety of ways (*n* = 8, 50% male), jumping in different ways (*n* = 6, 50% male), or standing (*n* = 6, 50% male) on a ladder in a pool or lake area. The photos were obtained through an online images search, with similar images included to show boys and girls equally often and in the same situations. In addition to the photos, three clipart images of a figure diving into water were included and randomized within the deck, with different types of dives represented (see Figure 1); these clipart images were chosen to match images commonly depicted on No Diving warning signs.

#### 2.2.2. Warning Signs and Related Materials

Materials were developed to assess children’s understanding of text and images commonly found on No Diving warning signs. All warning signs created were constructed to have large, bold text with red lettering on white background, which is a color combination often used on warning signs [13].

**Understanding Text.** To assess children’s understanding of the signal safety words Danger, Caution, Warning they were asked to explain the meaning of the word that was printed on an index card. These cards were presented one at a time, randomly interspersed between other questions.

To assess children’s understanding of why one should not dive if there is shallow water, they were asked to explain a sign indicating No Diving—Shallow Water (i.e., there were no images, just text on this sign) and to say what could happen if they ignored the sign and dove into a pool having shallow water.

To assess children’s understanding of common phrases that are used on warning signs to communicate about the consequence of diving into shallow water children were sequentially presented (randomized) two signs that said No Diving—Shallow Water and then under this said either You could break your neck or You can become permanently injured, and they were asked to explain what each phrase meant. Note that we purposefully embedded the consequence phrases within the context of a No Diving warning sign because our aim was to determine how children interpret these phrases when they see them under typical warning circumstances.

**Figure 2:**
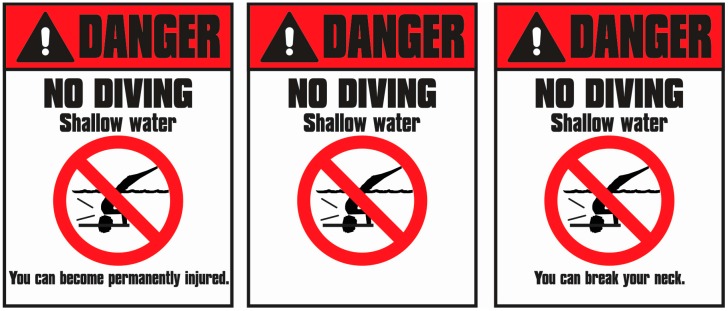
The warning signs shown to children individually to assess their understanding of the image (**center**); the phrase “permanently injured” (**left**); and the phrase “break your neck” (**right**).

**Understanding Images.** To explore children’s understanding of a common clipart image used on no diving warning signs, they were presented a sign that said Danger! No Diving—Shallow Water that had a clipart image showing a broken neck but no interpretive text, and asked to explain what the image was telling them (see middle sign in Figure 2). Note that we purposefully embedded the image within the context of a no diving warning sign because our aim was to determine how children interpret the image when it is presented within a warning sign context.

### 2.3. Structured Interview

Children were interviewed individually in a quiet room, with interviews tape recorded for later transcribing and coding. Children started with an index card (randomized order) and were asked what the safety signal word meant; they were given up to one prompt if they indicated they did not know or said something unclear. Children were then shown the No Diving—Shallow Water sign, and asked what the sign would be trying to tell them if it was hanging near a pool and then to comment on what could happen if they ignored the sign and dove into a pool having shallow water; again, up to one prompt was allowed; children answered questions with reference to themselves, not all children in general. The researcher then asked the child to look at each one of the photos they had put in the Diving box and to rate each one for how dangerous it would be to do the dive shown in the picture into a pool having shallow water (0 = not at all, 1 = a little, 2 = fairly, 3 = very, 4 = extremely dangerous). These scores were then averaged to create one score for each child for danger of diving into shallow water. Children were asked to rate both how likely it would be to get hurt from diving into a pool having shallow water (0 = not at all, 1= a little, 2= fairly, 3 = very likely, 4= for sure they would get hurt), and to rate the severity of the possible injury (0 = not serious, 1= somewhat, 2 = fairly (e.g., broken bone), 3 = very serious, such as hospitalized, 4 = death). The children were shown a No Diving—Shallow Water sign with a clipart image of a character diving into shallow water and breaking their neck (see middle sign in Figure 2) and they were asked to explain what is happening in the image depicted and what the image was trying to tell them. They were then shown (random order) each of the other two signs from Figure 2, one at a time, and were asked to explain what the phrase means (permanently injured, you can break your neck). The child was then asked to judge whether it is possible to end up in a wheelchair for life from diving into shallow water (yes, no).

### 2.4. Procedure

The parent and their child visited a laboratory on campus for a one hour session. After signing consent forms, the parent filled out questionnaires (family demographics, child’s history of swim lessons and experience diving) while the child went into another room with a research assistant. For the photo sort task, children were given a randomized ordering of photos and asked to sort these into two boxes—one for Diving photos and the second for Not Diving photos. The structured interview then was completed, with this audio recorded for later transcribing and coding. At the conclusion of the session, the child was thanked, reminded about safety practices near water, and given a gift card for participating.

### 2.5. Data Coding

For diving status, children were assigned a score based on whether they did or did not have experience (score of 1 or 0, respectively). Responses to open-ended questions were transcribed and then coded using a scheme developed for each question individually, with higher scores indicating greater understanding. Children’s understanding of the terms Danger, Warning, and Caution were coded as: 0 = no clear understanding; 1= understanding that it is indicating something you should not do; 2 = understanding that you should not do it because of physical injury. Answers to the question of what a “No Diving—Shallow Water” warning sign means were coded as: 0 = no clear understanding or answer has nothing to do with safety (e.g., mentions getting in trouble); 1 = understands that one should not dive because of safety but does not clearly state why; 2 = clear understanding that one should not dive because the shallow water makes it unsafe and likely to get hurt. Answers to what could happen if one ignored the sign and dove into a pool with shallow water were coded as: 0 = answer has nothing to do with safety (e.g., mentions getting in trouble); 1 = answer that reflects a “can get hurt” idea; 2 = mentions a specific way one could get hurt by hitting the bottom (e.g., bang your head, smash your face, hit your hands); 3 = mentions specific possible consequence of hitting the bottom (e.g., get a concussion). Responses for their level of understanding of the consequence phrases (permanent injury, break your neck) were coded as: 0 = no clear understanding, 1 = answer indicating being seriously hurt and hospitalized, 2 = clear understanding that effects involve limitations in movement and are lifelong (e.g., mentions paralysis or not being able to walk or move independently). Children’s responses about what was happening in the clipart image that appeared on the warning sign (see middle sign in Figure 2) were coded as: 0 = no clear understanding, 1 = mentions banging head or face on bottom, 2 = mentions injury to face, head, or neck; 3 = specifically mentions broken neck.

A second coder independently scored the data from 25% of the sample, with reliability for agreement in coding reaching 92% (kappa = 0.91) minimally. The data of the primary coder were analyzed.

### 2.6. Analytic Approach

Descriptive and parametric statistics were applied to characterize the data and evaluate the pattern of results as a function of age group, gender, and diving experience. Several preliminary data checking procedures were applied before analyses were conducted, including screening for outliers using Cook’s distance and checking that variables were normally distributed [25]; no issues were noted. Before reporting within-participant Analysis of Variance (ANOVA) results we assessed for violations of sphericity to determine if adjustment to the degrees of freedom was warranted, in which case a Greenhouse-Geisser adjustment was used. Effect sizes are reported as partial eta squared. In conducting paired contrasts using *t*-tests, a Bonferroni adjustment for family-wise error rate was applied; the results reported are based on this adjustment having been applied.

### 2.7. Ethical Approval

Prior to initiating this research, the study protocol was reviewed by, and approval was obtained from, the Research Ethics Board committee at the University of Guelph (13MY009). Participants and their custodial parent granted written consent prior to participation in this study.

## 3. Results

Both age and diving experience were of interest, however, because these variables were positively correlated (*r* = 0.32, *p* < 0.01), these factors were not considered simultaneously in the same analyses. Rather, in executing the ANOVAs we assessed for each separately while controlling for the effects of the other variable. In addition, gender differences were examined, where appropriate.

### 3.1. Judgments of What Qualifies as “Diving”

Despite the variation in types of dives portrayed in the photos and clip art images (e.g., deep dives, shallow dives, jackknife dives), children correctly identified “diving” photos and images with nearly perfect accuracy, 98%. Thus, children are aware of the variety of forms that diving can take and judge that all of them would qualify if one is referring to “diving”, such as in a warning sign.

### 3.2. Understanding of Signal Words (Danger, Warning, Caution)

To test for differences in children’s understanding of the terms *Danger*, *Warning*, and *Caution* and determine factors that affected this understanding, a mixed ANOVA was conducted with age group (2: younger, older) and gender (2: boy, girl) as between-participant factors and signal word type (3: *Danger*, *Warning*, *Caution*) as a within-participant factor, with diving experience entered as a covariate. Results revealed a significant main effect of age group, *F* (1, 115) = 10.99, *p* < 0.05, η_p_^2^ = 0.08. Although all children understood that the terms were alerting you to something you should not do (score of 1), older children demonstrated a better overall understanding of the signal terms compared to younger children (*M =* 1.22 and 1.00, *SD* = 0*.*05 and 0.06, respectively). There also was a significant main effect of signal word, *F* (2, 230) = 24.41, *p* < 0.05, η_p_^2^ = 0.18. Follow-up paired-comparison t-tests revealed that children’s understanding of *Danger* significantly exceeded their understanding of the terms *Warning* and *Caution* (*M* =1.49, 0.97, and 1.02; *SD* = 0.58, 0.54, and 0.52, respectively; *t* (119) = 8.11 and *t* (119) = 9.20, *p* < 0.001, respectively), which did not differ from one another, *p* > 0.05. There were no significant differences related to gender, *p* > 0.05.

To explore if diving experience affected children’s understanding of these signal words, a mixed ANOVA was conducted with diving experience (2: yes, no) and gender (2: boy, girl) as between-participant factors and signal word type (3: *Danger*, *Caution*, *Warning*) as a within-participant factor, with age group entered as a covariate. Results revealed an interaction of diving experience with signal word, *F* (2, 230) = 3.49, *p* < 0.05, η_p_^2^ = 0.08; there were no significant effects related to gender, *p* > 0.05. Three independent t-tests comparing experienced and inexperienced divers’ understanding of each signal word revealed, as shown in Table 1, diving experience was associated with greater understanding for *Danger* (*t* (89) = 7.07, *p* < 0.001), but it did not enhance children’s understanding of *Warning* or *Caution*, *p* > 0.05.

### 3.3. Understanding of a “No Diving—Shallow Water” Sign

To explore children’s understanding of what a “*No Diving—Shallow Water*” warning sign is trying to tell viewers, an ANOVA was conducted with age group (2: younger, older) and gender (2: boy, girl) as between-participant factors, with diving experience entered as a covariate. Similarly, an ANOVA was conducted with diving experience (2: yes, no) and gender (2: boy, girl) as between-participant factors, with age group entered as a covariate. Results of both ANOVAs revealed no effects of age, gender, or diving experience. Children understood the sign to mean that one should not dive because doing so was unsafe because of shallow water (*M* = 1.95, *SD* = 0.05).

### 3.4. Injury Risk-Appraisals Related to Diving

To explore children’s appraisals of injury risks related to diving in shallow water, their ratings of dangerousness, likelihood of being injured (injury vulnerability), and potential severity of injury were examined. In each case, an ANOVA was conducted with age group (2: younger, older) and gender (2: boy, girl) as between-participant factors, with diving experience entered as a covariate. There were no significant effects due to child age and/or gender, *p* > 0.05. Children rated diving into a pool having shallow water as being *very* dangerous (score of 3; *M* = 2.93, *SD* = 0.59), *very* likely to lead to injury (score of 3; *M* = 2.98, *SD* = 0.40), and likely to lead to a *very* serious injury that would require hospitalization (score of 3; *M* = 3.17, *SD* = 0.45).

To explore if diving experience affected these appraisals of injuries, a separate ANOVA was conducted for each appraisal with diving experience (2: yes, no) and gender (2: boy, girl) as between-participant factors and age entered as a covariate. The only effect was for diving experience, *F* (1, 115) = 3.72, *p* < 0.05, η_p_^2^ = 0.03. Children with diving experience judged their risk of injury to be significantly *lower* than those without diving experience (*M* = 2.89 and 3.23, *SD* = 0.94 and 0.73, respectively).

### 3.5. Understanding of What Could Happen From Diving into Shallow Water

***Text Based Messages***. First, children’s scores for what could happen if they ignored a “No Diving—Shallow Water” warning sign and dove into a pool were analyzed. An ANOVA was conducted with age group (2: younger, older) and gender (2: boy, girl) as between-participant factors, and diving experience entered as a covariate. Results revealed a significant main effect of age group, *F* (1, 115) = 4.44, *p <* 0.05, η_p_^2^ = 0.04. Older children provided more detailed responses about consequences associated with diving into a shallow pool than younger children (score of 1 = get hurt and 2 = get hurt *by* hitting the bottom; *M* = 1.94 and 1.65, *SD* = 0.69 and 0.66, respectively). There were no effects related to gender, *p* > 0.05. An ANOVA with diving experience (2: yes, no) and gender (2: boy, girl) as between-participant factors and age entered as a covariate, revealed no significant effects related to diving experience, *p* > 0.05.

Second, children were asked the meaning of the term “*Permanently Injured*”*.* An ANOVA was conducted with age group (2: younger, older) and gender (2: boy, girl) as between-participant factors, and diving experience entered as a covariate. Results revealed a significant main effect of age group (*F* (1, 115) = 5.04, *p <* 0.05, η_p_^2^ = 0.04), indicating that older children had a better understanding of the term than younger children (score of 1 = seriously hurt and 2 = lifelong effects, *M* = 1.95 and 1.41 and, *SD* = 0.44 and 0.49, respectively). There were no effects related to gender, *p* > 0.05. An ANOVA with diving experience (2: yes, no) and gender (2: boy, girl) as between-participant factors and age as a covariate revealed no significant effects.

Children’s understanding of the term “*Break Your Neck*” also was assessed. An ANOVA was conducted with age group (2: younger, older) and gender (2: boy, girl) as between-participant factors, and diving experience entered as a covariate. Similarly, an ANOVA was conducted with diving experience (2: yes, no) and gender (2: boy, girl) as between-participant factors and age entered as a covariate. The results of these ANOVAs revealed no effects of child age, diving experience and/or gender on their understanding of the term, *p* > 0.05. Children understood the phrase to mean that there would be limitations in movement that lasted for life (score of 2; *M* = 2.08, *SD* = 0.09). Consistent with this, when asked whether or not it was possible to wind up in a wheelchair for life from diving into a pool, 78% agreed with the statement and Chi Square tests revealed no differences related to age or diving experience, *p* > 0.05. When these children were asked how they had learned about the dangers of diving into a pool having shallow water, 47% reported learning this on their own based on pool experiences and the remaining 53% reported being taught this through swim lessons. Chi Square tests indicated that these percent scores did not vary with age group or diving experience, *p* > 0.05.

***Images****.* Children’s understanding of the clipart images was also examined. An ANOVA was conducted with age group (2: younger, older) and gender (2: boy, girl) as between-participant factors, with diving experience entered as a covariate. Similarly, an ANOVA was conducted with diving experience (2: yes, no) and gender (2: boy, girl) as between-participant factors and age entered as a covariate. Results of both ANOVAs revealed no significant effects, *p* > 0.05. Regardless of age, gender and/or diving experience, children interpreted the cartoon image as showing that diving will lead to hitting your head on the bottom of the pool (score of 1.0; *M* = 1.10, *SD* = 0.36).

## 4. Discussion

Warning signs are intended to alert viewers to hazards and risk behaviors to avoid. Although these are assumed to be equally effective across a broad age range, the majority of research on this medium has focused on adults. Much less is known about children’s understanding of warning signs. Moreover, despite the fact that diving is a risk factor for spinal cord injury even in children [10], there is limited systematic research that specifically addresses their understanding of No Diving warning signs. Addressing these gaps, the current study examined children’s understanding of common image and text features that are included on No Diving warning signs. The results provide important insights into children’s understanding of injury-risk information about diving communicated on warning signs, as well as how this understanding varies as a function of child gender, age, and diving experience.

With regard to images, the fact that children categorized clipart images showing diving in the same way they did actual photos confirms that they have no difficulty understanding the representational diving behavior depicted in the clipart images used on warning signs. Moreover, despite variation in the type of dives depicted (shallow, deep, jackknife, etc.), children categorize them all as “dives”. This is an important finding because it indicates that children generalize across different exemplars to understand that “diving” refers to many different types of head first entries into a pool. Therefore, it is less important as to what specific diving image is shown, as long as the word “diving” appears on the warning sign with the image.

Children also were asked to explain what the diving image on the warning sign was communicating. The majority interpreted the image as indicating that diving will lead to hitting your head on the bottom of the pool. However, they generally did not interpret the image as depicting someone breaking their neck from doing so. Thus, this very popular image on No Diving signs is not effective *on its own* to alert children to the risk of permanent (i.e., break your neck) injury. However, when the image is placed in the broader context of text-based information on a No Diving warning sign and the totality of our results are considered, the results indicate that children 7 to 10 years understand the dangers and potential seriousness of consequences from diving into shallow water.

With regard to text-based messaging, children understood that all three popular signal words are communicating something one should not do. Nonetheless, they showed the greatest understanding that there was a safety issue when the term *Danger* was used*.* Thus, *Danger* should be the signal word of choice for warning signs targeting children. When presented a sign saying “No Diving—Shallow water”, children also correctly interpreted the text to mean that one should not dive because doing so was unsafe. Although they did not always elaborate to explain how the shallow water made it unsafe, ratings of their injury risk appraisals revealed they understood that diving into a pool having shallow water was *very* dangerous, *very* likely to result in injury, and that this injury could be *very* serious and require hospitalization.

Despite this knowledge and understanding of diving-related injury risks, it is noteworthy that the majority of children did not *spontaneously* mention permanent and catastrophic effects when asked about what could happen from diving in shallow water. Rather, they discussed consequences in more general terms (e.g., getting hurt from hitting the bottom). Nonetheless, when questioned directly, children’s responses indicated that knew that “breaking your neck” would result in limitations in movement that lasted for life, and the majority agreed that one could wind up in a wheelchair for life from diving into shallow water. The pattern of these results suggest that children may not routinely anticipate or consider that permanent and catastrophic injury is likely to result from diving into shallow water, but they understand that this can occur. Explicitly saying on No Diving signs that one can “break your neck” may bring this knowledge to consciousness *in the situation*, thereby maximizing effectiveness of the warning to deter diving by children. In addition, providing active supervision by adults has been shown to reduce children’s risk behaviors [26,27] and also may increase the likelihood that children comply with the warning, act on the knowledge they have, and avoid diving into shallow water. Given that behaving in unexpected ways is common during childhood and a risk factor for injury [28,29], active supervision is essential in water-related risk situations where children can be seriously injured.

Importantly, this research also provides insights regarding whether there are differences in children’s understanding of No Diving warning signs that relate to their gender, age, and past diving experience. There were no significant differences in boys’ and girls’ understanding of No Diving warning signs. Although boys typically engage in more risk behaviors and experience more injuries than girls, including diving injuries [1,5,9,30], the current findings suggest that their level of understanding of warning signs prohibiting diving and communicating risks from doing so is comparable to that of girls. Therefore, given a diving risk context in which warning signs are posted, gender differences in diving rates are not due to differential understanding of the risks of doing so. Rather, gender differences in diving injury statistics may reflect differential effectiveness of warning signs and relate to differences in boys’ and girls’ injury-related attitudes and beliefs [31,32]. Past research on predicting children’s risk practices has shown that girls focus on vulnerability for injury (“can I get hurt doing this”), whereas boys focus on potential severity of injury (“how badly hurt could I get hurt”). Warning signs that communicate a risk of injury, therefore, may be sufficient to deter girls from diving, but boys would presumably need messaging that emphasizes the potential consequences of diving. Thus, explicitly saying on No Diving signs that one can “break your neck”, may be especially important to deter diving by boys.

There were a few differences in results related to children’s age. Children at older ages showed a better understanding of the signal words than younger children, although all understood these were alerting you to something you should not do. In addition, older children more frequently than younger children reported that the term “permanent injury” means a lifelong effect, although all children understood that it was referring to a severe injury. Finally, although younger children understood that it was dangerous to dive, this was prohibited, and doing so could result in a very serious injury requiring hospitalization, they were less likely than older children to interpret the text information about shallow water as indicating a concern about hitting the bottom of the pool. However, they did interpret the clipart image as warning about this consequence of diving (see above). Thus, although there were some age differences that emerged when examining selected aspects of the data, when one considers children’s comprehensive understanding of No Diving warning signs that comprise both image *and* text-based information, the results suggest that younger children’s understanding of diving in shallow water is comparable to that shown by older children (very dangerous, can hit the pool bottom, very likely to lead to injury, injury can be very serious and require hospitalization).

Diving experience biased children toward assuming less likelihood of injury. This finding is consistent with other studies that have found that experience with an activity predicts greater risk taking because children develop an optimism bias and downplay warnings about injury risk, assuming that risk behaviors pose less risk of injury for them than less experienced peers [32,33,34]. Thus, despite rating diving into shallow water as *very* dangerous and likely to lead to a *very* serious injury that would require hospitalization, children with diving experience expected their personal risk of injury to be lower than those not having diving experience. No Diving warning signs, therefore, are likely to be less effective for those children who have past experiences successfully engaging in the risk behavior; similar effects of past experience have been noted in research on warning sign effectiveness with teens and adults [21,23,35]. Active supervision by adults, therefore, will be particularly important to prevent children who have prior experience diving from attempting to do so despite the presence of warning signs prohibiting the behavior.

Finally, although this study focused on warning signs, it is not our intention to suggest that this is necessarily the best or only strategy for preventing diving-related injuries. Without doubt, maximizing reductions in these injuries will require a comprehensive and multi-faceted approach. For example, integrating active supervision with signage and enforcement strategies (e.g., penalties for non-compliance with signage or pool rules), and coupling this with environmental modifications (e.g., pool designs) that reduce risk of serious injury or prevent risk behaviors (e.g., designing above-ground pool ladders to prevent diving), addresses many significant risk factors for diving injuries.

### Limitations and Future Research

Although the current study provides insights into what children understand about diving into shallow water from warning signs, there are some limitations that merit discussion and consideration in planning future research. First, the sample in the study was fairly homogeneous and comprised predominantly Caucasian, middle income, and well educated families. A more diverse sample would be desirable in future studies because exposure to factors that could influence children’s understanding of diving risks (e.g., recreational water experiences, news stories/discussion about diving injuries) would be expected to vary with income and parent education levels.

Second, nearly all the children in this sample had participated in formalized swim lessons, however, reliable information on the extent of swim lesson experience could not be obtained. This precluded our examining the extent to which swim lesson experience influences children’s understanding of diving risks. In future research, therefore, it would be useful to gather more systematic information on the nature and extent of swimming lesson experiences and to determine how these experiences influence children’s understanding of the text- and image- based messages communicated in No Diving warning signs. Findings from such research could provide important information on aspects of swim and/or diving instruction that merit changing.

Finally, results suggest that prior experience is a risk factor for diving even in the presence of a warning sign cautioning against doing so: children with experience rated their likelihood of injury from diving as lower than did children without such experience even though there were no group differences in ratings of the extent of dangerousness or severity of potential injury. We did not, however, measure the nature or extent of past diving experience to determine if differences emerge related to these factors. It could be, for example, that one needs experience in the same situation (e.g., diving in a similar type of pool) before an optimism bias emerges about injury risk. Or, that the extent of optimism bias is positively related to the extent of past successful diving experiences. These are important relations to examine because the findings could have aide in identifying which children are at greatest risk of ignoring a No Diving warning sign and, therefore, in greatest need of active supervision.

## 5. Conclusions

The results of this study on No Diving warning signs revealed that children understand the behavior advised against (i.e., diving), why it is prohibited (i.e., can hit head on the bottom), and what can happen from doing the behavior (i.e., serious injury that may require hospitalization). They knew that if you “break your neck” this would result in limitations in mobility for life and the majority agreed that diving can result in being in a wheelchair for life. Nonetheless, children do not routinely consider that permanent injury can result from diving into shallow water. Explicitly saying on No Diving signs that one can “break your neck” may improve the effectiveness of signs by reminding children in the risk situation what they know, namely—that there can be severe and life-long consequences of diving. The results also suggest that children with past successful diving experience comprise a high-risk group that may be less likely to comply with warning signs. Active supervision is essential for all children in drowning- and diving-risk situations, but is particularly important to ensure the safety of children with prior positive diving experiences as this seems to constitute a “high risk” group.

## Figures and Tables

**Table 1:** Extent of understanding of signal words (possible range: 0–2.0) as a function of diving status.

Diving Status	Signal Word ^1^		
	Danger ^2^	Warning	Caution
Experienced	1.67 (0.55)	0.83 (0.53)	0.87 (0.59)
Inexperienced	1.43 (0.58)	0.98 (0.56)	0.91 (0.55)

**Notes: ^1^** Danger > Warning = Caution, *p* < 0.05; **^2^** Experienced > Inexperienced, *p* < 0.05.
